# A look at the diastolic function in severe sepsis and septic shock

**DOI:** 10.5935/0103-507X.20150052

**Published:** 2015

**Authors:** Vicente Cés de Souza Dantas, Eduardo Leite Vieira Costa

**Affiliations:** 1 Universidade Federal do Rio de Janeiro - Rio de Janeiro (RJ), Brazil.; 2 Intensive Care Unit Respiratory, Discipline of Pulmonology, Instituto do Coração, Hospital das Clínicas, Faculdade de Medicina, Universidade de São Paulo - São Paulo (SP), Brazil.

Myocardial dysfunction in sepsis is a complex entity due to the dynamic adaptation of the cardiovascular system to the disease process, the host response, and the effects of resuscitation. The pathophysiology of this entity is multifactorial; systemic, cellular, and extracellular mechanisms have been described, including maldistribution of coronary blood flow, myocardial injury, complement-triggered (C5a) myocyte contractile failure, cytokine-induced neutrophil activation (tumor necrosis factor, interleukin-1β, interleukin-6), dysregulation of calcium handling, and cytopathic hypoxia due to mitochondrial dysfunction.^(1,2)^

Cardiovascular compromise is a central component of multiple organ dysfunction syndrome, an often fatal consequence of severe sepsis and septic shock. There is a sizable body of work on left ventricular systolic dysfunction in sepsis,^(3,4)^ whereas other forms of myocardial dysfunction have been often overlooked. These variants include left ventricular diastolic dysfunction^(5-8)^ and right ventricular dysfunction,^(9,10)^ which have different treatment options and prognostic implications. Interestingly, all types of myocardial dysfunction can be present in isolation or in combination and can be fully reversible with resolution of critical illness.^(5,11)^

With the use of tissue Doppler imaging (TDI), a technique that has gained acceptance amongst cardiologists and intensivists for the evaluation of diastolic function, it has become evident that diastolic dysfunction is indeed common in critically ill patients.^(12)^ TDI is an echocardiographic technique based on measurements of myocardial velocities,^(13)^ which are low frequency, high-amplitude signals filtered from conventional Doppler imaging.^(14)^It is particularly useful as a measure of ventricular relaxation and ventricular filling pressures.^(15)^ The significance of diastolic dysfunction was recently highlighted by studies that demonstrated that TDI might be prognostically useful in the general intensive care unit (ICU) population.^(7,16-22)^

In this issue of the *Revista Brasileira de Terapia Intensiva*, an interesting, single-center prospective cohort study confirmed the importance of diastolic dysfunction in patients with severe sepsis and septic shock. The authors studied 53 patients with the aim of assessing the prevalence of myocardial dysfunction both at ICU admission and one week later and also of evaluating the impact of left ventricular systolic and diastolic dysfunction on ICU mortality.^(23)^

A detailed echocardiographic examination was performed within 48 hours of ICU admission and 7-10 days later, which included M-mode, two-dimension, and Doppler echocardiography. Systolic and diastolic function, including TDI, as well as right ventricular assessment with tricuspid annular plane systolic excursion, were carefully evaluated by a single cardiologist specialized in echocardiography.

The authors found an alarmingly high prevalence of diastolic dysfunction of 84% in the group of septic patients, a finding that remained unaltered in the follow-up echocardiography performed one week later. This high prevalence might be related to the age range (74 ± 13 years) of patients included. However, we cannot exclude the possibility of overestimation due to selection bias because only 25% of eligible patients entered the study with a comparable number of patients being lost to follow-up. Additionally, although tempting, we cannot ascribe all the echocardiographic findings to septic cardiomyopathy. Diastolic dysfunction could as well be a premorbid condition in the sample of elderly patients studied, a hypothesis supported by the lack of resolution of echocardiographic findings in the follow-up exam.

Another relevant finding was the association of diastolic dysfunction with mortality, confirming^(23)^ the importance of this previously overlooked variant of myocardial compromise. The E/e' ratio, one of the TDI measures used to define diastolic dysfunction, was the only echocardiographic measurement independently associated with death even after adjusting for age and disease severity. Interestingly, systolic dysfunction was not a predictor of death. These findings highlight the need to perform thorough echocardiographic evaluations in patients with sepsis and septic shock.

In his famous work *The Unbearable Lightness of Being*, Milan Kundera was right to acknowledge the powerful predictive ability of the heart: "When the heart speaks, the mind finds it indecent to object".^(24)^
